# Non-addictive opium alkaloids selectively induce apoptosis in cancer cells compared to normal cells

**DOI:** 10.1186/s40199-015-0101-1

**Published:** 2015-02-20

**Authors:** Monireh Afzali, Padideh Ghaeli, Mahnaz Khanavi, Maliheh Parsa, Hamed Montazeri, Mohammad Hossein Ghahremani, Seyed Nasser Ostad

**Affiliations:** Department Toxicology & Pharmacology, Faculty of Pharmacy, Toxicology & Poisoning Research Center, Tehran University of Medical Sciences, 14155/6451 Tehran, Iran; Department Clinical Pharmacy, Faculty of Pharmacy & Rational Drug Use Research Center, Tehran University of Medical Sciences, 14155/6451 Tehran, Iran; Department Pharmacognosy, Faculty of Pharmacy, Tehran University of Medical Sciences, 14155/6451 Tehran, Iran

**Keywords:** Cancer cell, Cytotoxicity, Genotoxicity, Narceine, Noscapine, Papverine

## Abstract

**Background:**

Cytotoxic effects of some of the members of papaveraceae family have been reported in Iranian folk medicine. Recent reports has indicated that alkaloids fraction of opium may be responsible for its cytotoxic effect; however, the mechanism of this effect is not fully understood. This study has been designed to investigate the selective cytotoxic, genotoxic and also apoptosis induction effects of noscapine, papaverine and narceine, three non-addictable opium alkaloids, on HT29, T47D and HT1080 cancer cell lines. Mouse NIH3T3 cell line was chosen to present non-cancerous cells and Doxorubicin was selected as the positive control.

**Methods:**

Cells were treated by different concentrations of Noscapine, Papaverine, Narceine and doxorubicin; viability was assessed by MTT assay. The genotoxicity and apoptosis induction were tested with comet assay and Annexin-V affinity when the concentration of each these drugs is less than its IC50. In addition, the DNA damage and caspase activity of the T47D cells were examined and the results were compared.

**Results:**

This study noted the cytotoxicity and genotoxicity of noscapine and papaverine, specifically on cancerous cell lines. Furthermore, papaverine induces apoptosis in all studied cancer cell lines and noscapine showed this effect in T47D and HT29 cells but not in NIH-3 T3 cells as noncancerous cell line. narceine also showed genototoxicity in the studied cell lines at its IC50 concentration.

**Conclusions:**

This experiment suggests that noscapine and papaverine may be of use in cancer treatment due to their specific cytotoxicity and genotoxicity. However, further in vivo studies are needed to confirm its usefulness in cancer treatment.

## Background

The agents which have the potential for induction of apoptosis may be considered as good candidates for cancer therapy due to their effect on the uncontrolled proliferation of malignant cells. In spite of excellent anti-tumor activities of common chemotherapy drugs, treatment will be restricted in some cases due to drug-resistance, low therapeutic index, severe side effects and different routes of administration [1]. Recently, in search for new treatments for cancer, there has been an emphasis on herbal and natural compounds. Cytotoxic effects of some members of Papaveraceae family have been considered in Iranian and Indian medicine [2]. *Papaverine somniferum L.* (opium poppy) has been traditionally used in Chinese and Indian herbal medicine to cure some disorders including chronic cough, dysentery, diarrhea, rectum prolapse and gastrointestinal problems [3]. The contents of opium alkaloids include morphine, codeine, tebaine, noscapine, papaverine, narceine as well as little percentage of some other compounds [4]. Many studies revealed a remarkable anti-tumor activity of the alkaloid noscapine, a naturally-occurring benzylisoquinoline alkaloid that constitutes about 2-10% of the alkaloid content of opium [5,6]. Furthermore, the anti-tumor activities of noscapine and its tubulin-binding property have been mentioned in many studies [7-12]. Noscapine inhibits the progression of melanoma, lymphoma, leukemia, breast cancer, colon cancer, ovarian carcinoma, glioblastoma, non-small cell lung cancer and prostate cancer while it has a little or no significant toxicity to the kidney, heart, liver, bone marrow, spleen and small intestine [8,12-16]. Papverine and narceine are two other benzylisoquinoline alkaloids constituting 0.5-3% and <0.5% of the alkaloid contents of opium, respectively. Papaverine has been used clinically as smooth muscle relaxant, anti-spasmodic for gastrointestinal disorders, cough suppressant and for treatment of erectile dysfunction (an unlabeled use). Although cytotoxic effect of papaverine and some of its derivatives has been observed in breast cancer, melanoma and prostate cancer [17-20], their mechanism of action has not been fully understood. Based on our knowledge, no previous study has examined the anti-cancer activities of narceine.

In this study three different human cancer cell lines have been chosen. HT29 colorectal carcinoma and T47D breast cancer cells as two of the most common types of human tumors and HT1080 fibrosarcoma cells as one of the most resistant cell lines to anti-cancer therapy. In addition, non-cancerous NIH-3 T3 cell line has been chosen to compare the effect of the agents between cancerous and non-cancerous cells and doxorubicin has been chosen as positive control. The purpose of this study is to show whether papaverine and narcein have selective cytotoxicity, genotoxicity and induction of apoptosis in cancerous cell lines as has been demonstrated in noscapine and was claimed in the traditional medicine.

## Methods

### Cell lines and chemicals

T47D (Breast, ductal-carcinoma, Human), HT-29 (Colon, epithelial-like carcinoma, Human), HT-1080 (connective tissue, fibro sarcoma, Human) and NIH-3 T3 (Swiss mouse embryo fibroblast) cell lines were all obtain from National Cell Bank (Pasture institute of Iran, Tehran). The cells were cultured in RPMI 1640 medium (Biosera, England) that was supplemented with 10% heat-inactivated fetal bovine serum (FBS; Biosera, England) as well as antibiotic vials containing 100 U/ml penicillin and 100 μg/ml streptomycin (Gibco, USA); then were incubated in a humidified atmosphere with 5% CO_2_ at 37°C.

The materials used in the present study and the manufacturers provided them are as following: noscapine hydrochloride, agarose and low melting point agarose (LMP) from Sigma-Aldrich (USA), papaverine hydrochloride and ethanol from TEMAD Co. (Tehran, Iran), narceine trihydrate from Seqchem (UK), doxorubicin from Sobhan-daru (Tehran, Iran), MTT (3-[4,5-dimethylthiazol-2yl] -2,5-diphenyl tetrazolium bromide) powder and Caspase Assay fluorometric kit from Roche (Germany) and annexine V-PE apoptosis detection kit from Abcam (UK).

### In vitro cytotoxicity assay

The cells in logarithmic phase of growth were seeded into 96-well plates at a density of 10^4^ cells per well and allowed to incubate overnight at 37°C. Cells were treated with different concentrations of noscapine, papaverine, narceine and doxorubicin and were incubated for 48 hours and then cell viability was determined by MTT assay. Briefly, 25 μl of 5 mg/ml MTT solution in phosphate-buffered saline was added and incubated for four hours at 37°C. The medium was removed and 100 μl dimethylsulfoxide (DMSO) was added to each well. The formazan salts were quantified by reading the absorbance at a test wavelength of 570 nm and a reference wavelength of 690 nm [21-23]. The IC50 values were calculated from their cytotoxity dose–response curves.

### Single cell gel electrophoresis (SCGE)/comet

The treated cells were diluted with RPMI 1640 + 10% FBS to final concentration of 175000 cells/ml. Cells were treated with 0.4% methanol in the CO_2_ incubator for 3 hours. Each sample of 50 ul was harvested; then, immediately suspended in 450 μl of solution composed of 10 μl PBS and 75 μl 0.5% LMP agarose. The samples were then immediately coated on a uniform background of each rough microscope slide that had been previously prepared by 1% agarose. The agarose on the microscope slides was allowed to harden at 4°C for 10 minutes. After coating with supportive layer (LMP), slides were then placed in a chilled (4°C) lysis buffer (2.5 M sodium chloride, 100 mM EDTA, pH = 10, 10 mM Trise base, 1% sodium lauryl sarcosinate, 0.01% triton x-100) for 30 minutes to unwind the nuclear DNA at 4°C and drip dried. Slides were then immersed in the same buffer for 30 minutes at 24 V and 250 mA at room temperature. DNA damage was recognized by ethidium bromide and florescence microscope after neutralizing the gel with Tris buffer [24-26]. Pictures were captured electronically with an image analysis system and analyzed for fluorescence intensity. DNA damage was evaluated using the tail length and compared with the control group.

### Examination of PS exposure

Surface exposure of phosphatidyl serine (PS) by apoptotic cells was examined according to manufacturer’s protocol. Briefly, cells were seeded in 6-well plates (3 × 10^5^ Cells/well) and treated with the agents for 24 hours. Cells were collected and resuspended in binding buffer (10 mM HEPES/NaOH, pH 7.4, 140 mM NaCl, 2.5 mM CaCl_2_) then were incubated with Annexin V-FITC and PI for 15 minutes at room temperature and darkness. The fluorescent intensity of the cells was measured in FL1 (for FITC) and FL2 (for PI) using flow cytometry technique.

### DNA fragmentation

Following drug treatments, 2 × 106 cells were collected and incubated in lysis buffer in ice for 20 minutes. The cells were centrifuged at 4°C at 12000 g for 30 minutes and then the supernatant was extracted after being centrifuged using 1:1 mixture of phenol: chloroform. Two equivalences of cold ethanol with one-tenth equivalence of sodium acetate were added; nucleic acid contaminant was decanted and exposed to water-RNase solution for 30 minutes at 37°C. The samples were electrophoresed through 1.5% agarose gel that contained ethidium bromide at 5 V for 5 minutes and then the voltage increased to 100 V for an hour [27].

### Caspase activity assay

The caspase activity was investigated based on the Homogeneous Caspases Assay fluorimetric kit. According to the protocol, the cells were cultured in a “black microplate with clear bottom” and were treated. Caspase Substrate in lysis buffer was added and was then incubated at 37°C for 2 hours. The free substrate was determined fluorimetrically at 521 nm and the activity of caspases was quantified by a calibration curve.

### Statistical analysis

The results were analyzed using one way ANOVA followed by Tukey-Kramer Posttest. The p-value less than 0.05 (p < 0.05) was considered significant.

Any experimental research that is reported in this manuscript have been approved by ethics committee of pharmaceutical sciences research center of Tehran University of Medical Sciences.

## Results

### Cytotoxicity of noscapine, papaverine, narceine and doxorubicin on breast cancer, colorectal carcinoma and connective tissue fibro sarcoma cell lines and non-cancerous cell line

**Figure 1:**
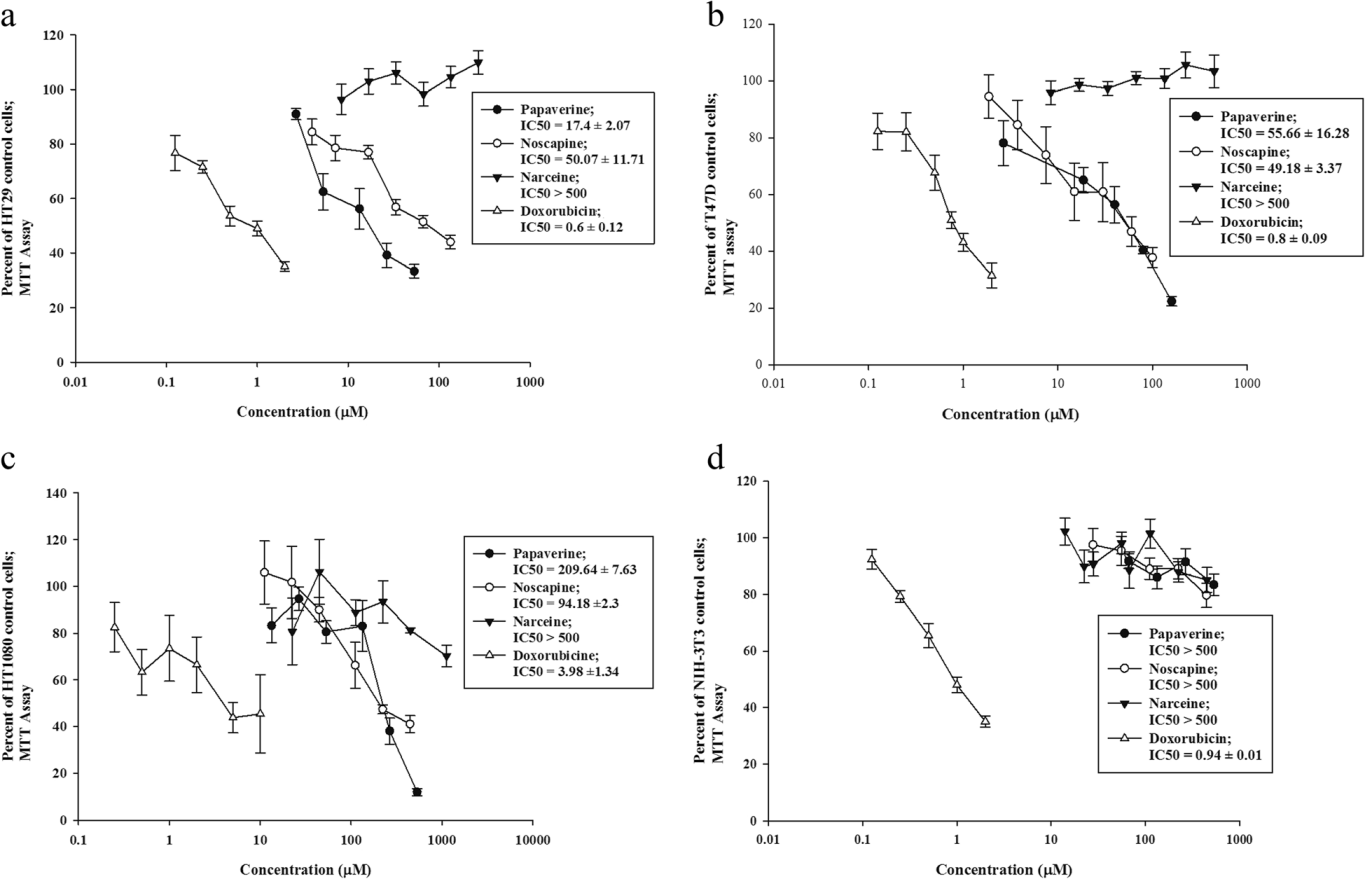
**Cytotoxic effects of opium alkaloids and doxorubicin on cell lines.** Cell viability in each treatment group was determined by MTT assay at 570 nm in reference standard at 690 nm. The IC50 values were calculated based on their cytotoxicity dose–response curve using Sigmaplot software and the results are expressed as Mean ± SD. **a**: HT29, **b**: T47D, **c**: HT1080 and **d**: NIH-3 T3.

The MTT curve showed that noscapine and papaverine had a dose-dependent cytotoxic effect on T47D, HT-29 and HT-1080 cell lines, with no cytotoxic effect on noncancerous NIH-3 T3 cells. Narceine did not show any toxic effect on the studied cell lines and doxorubicin-induced cytotoxicity was demonstrated on both cancerous and noncancerous cells. The results are presented in Figure 1.

### Effect of noscapine, papaverine and narceine on DNA damage

**Figure 2:**
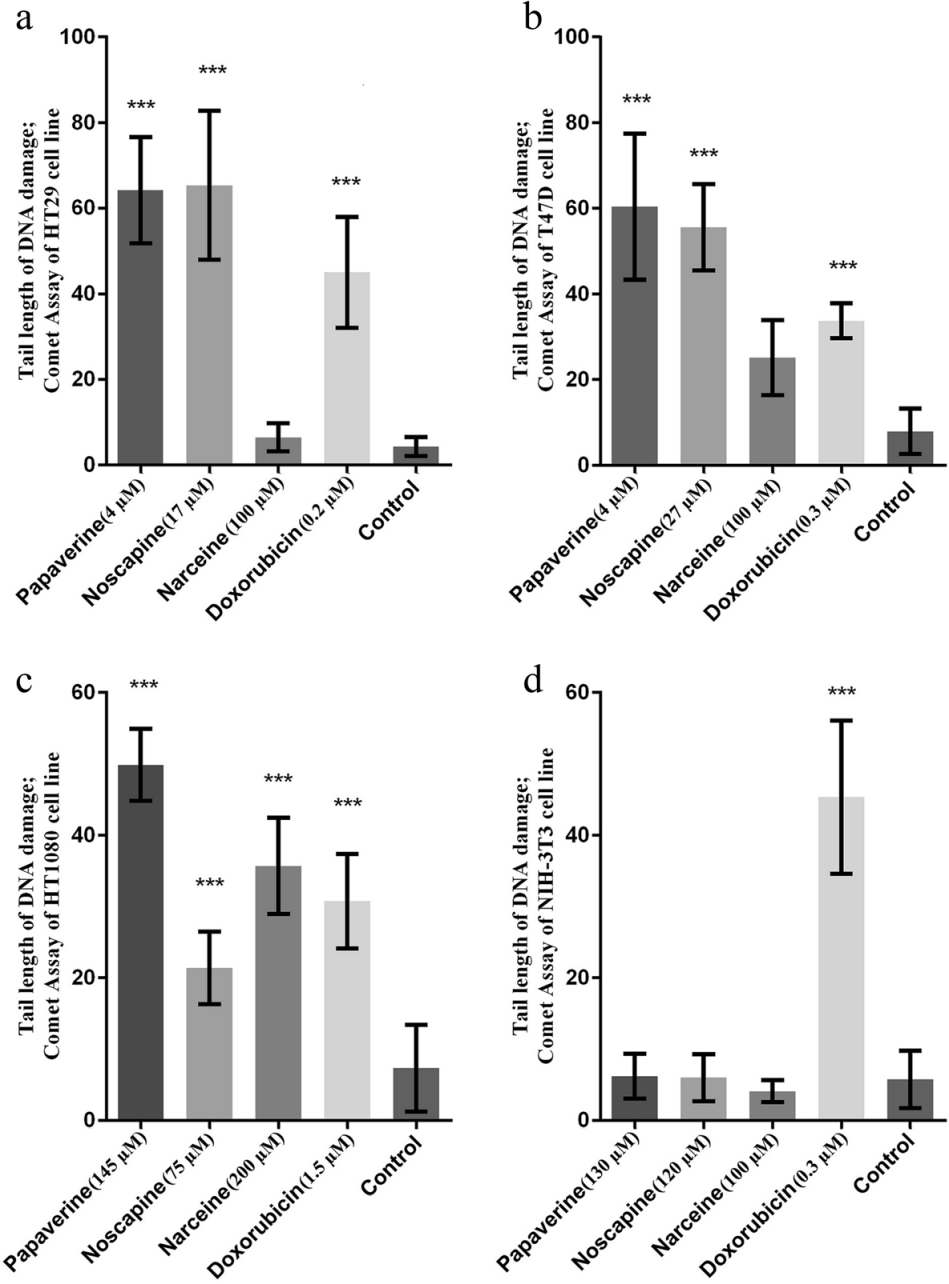
**The DNA damage detected in treated cells compared to control group.** The concentrations less than IC50 were used for treatment. The tail length of comet assay has been shown (Mean ± SD) and p-value has been reported. **a**: HT29, **b**: T47D, **c**: HT1080 and **d**: NIH-3 T3.

The cells were evaluated with an image analysis system (CASP Comet assay Software Project) and the results are expressed in terms of L tail (length of tail) and are presented in Figure 2. Noscapine and papaverine selectively enhanced DNA damage on cancerous cells when compared with noncancerous cells (p < 0.001). On the other hand, DNA damage was observed on all the cell lines after administration of doxorubicin (p < 0.001). However, narceine induced DNA damage only on HT-1080 cells.

### Effect of noscapine, papaverine, narceine on induction of apoptosis

**Figure 3:**
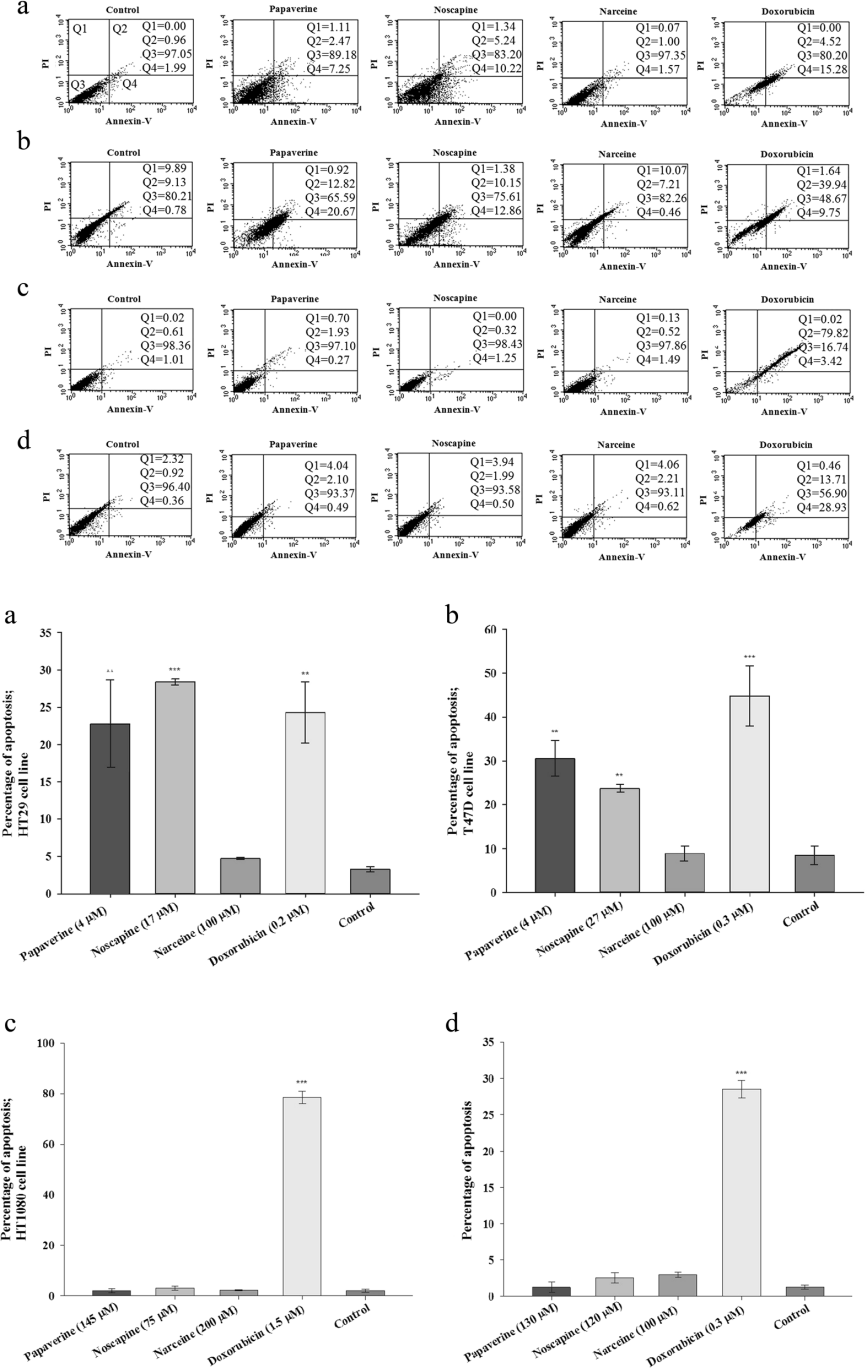
**The membrane asymmetry comparison between treated cells compared to control group.** The concentrations less than IC50 were used for treatment. The percentage of cell population of each quadrant has been shown and the quantitative average of percentage of apoptotic cells of repeated examinations (Mean ± SD) and p-value has been reported. **a**: HT29, **b**: T47D, **c**: HT1080 and **d**: NIH-3 T3.

Cell membrane asymmetry was investigated by translocation of phosphatidylserine to the cell surface in order to determine whether or not apoptosis was the major mechanism of cell death [28,29]. Based on this method, the apoptosis percentage has been reported and was compared with that in the control group (Figure 3). Results showed that doxorubicin has induced apoptosis in all the cell lines. However, noscapine and papaverine have induced apoptosis on HT-29 and T47D without any significant effect on NIH-3 T3 cell lines. However, the effects of narceine were different from the other two alkaloids. Narceine showed no apoptotic effect on the cell lines.

### DNA fragmentation in T47D cells treated by noscapine, papaverine, narceine and compared to doxorubicin

**Figure 4:**
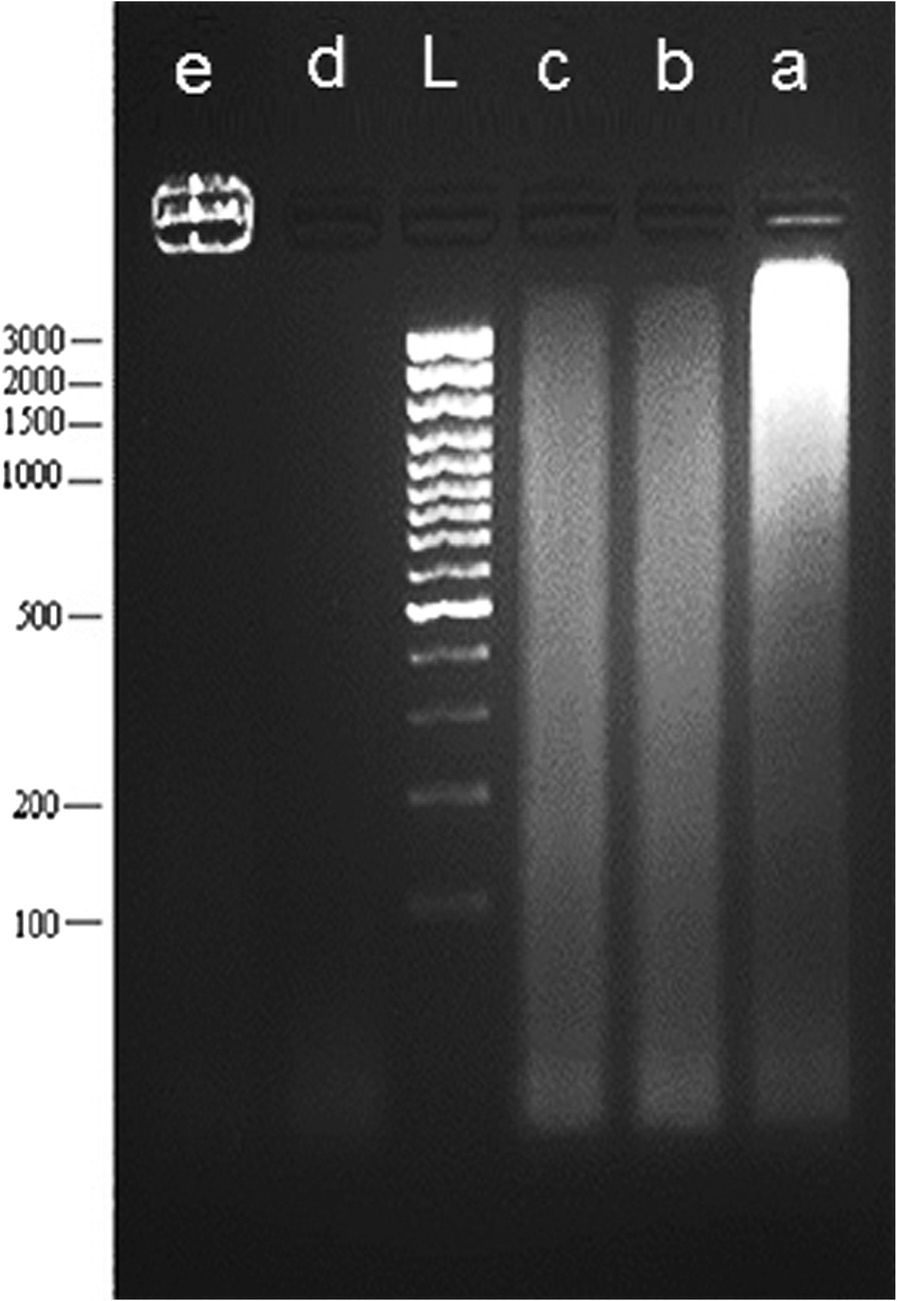
**Internucleosomal fragmentation of T47D cells.** The cells were treated with a: doxorubicin, b: noscapine, c: papaverine and d: narceine then DNA laddering was measured after 48 hours. e: negative control and L: Laddering marker are presented in figure as well.

To further determine if apoptosis was the major mechanism of the drug-induced cell death, DNA fragmentation was investigated in T47D cells. Figure 4 shows clear DNA fragmentation in cells treated by doxorubicin as positive control. Noscapine and papaverine induced DNA fragmentation in smaller sizes which were distinguishable from negative control group.

### Caspase activity in T47D cells treated by noscapine, papaverine, narceine and compared to doxorubicin

**Figure 5:**
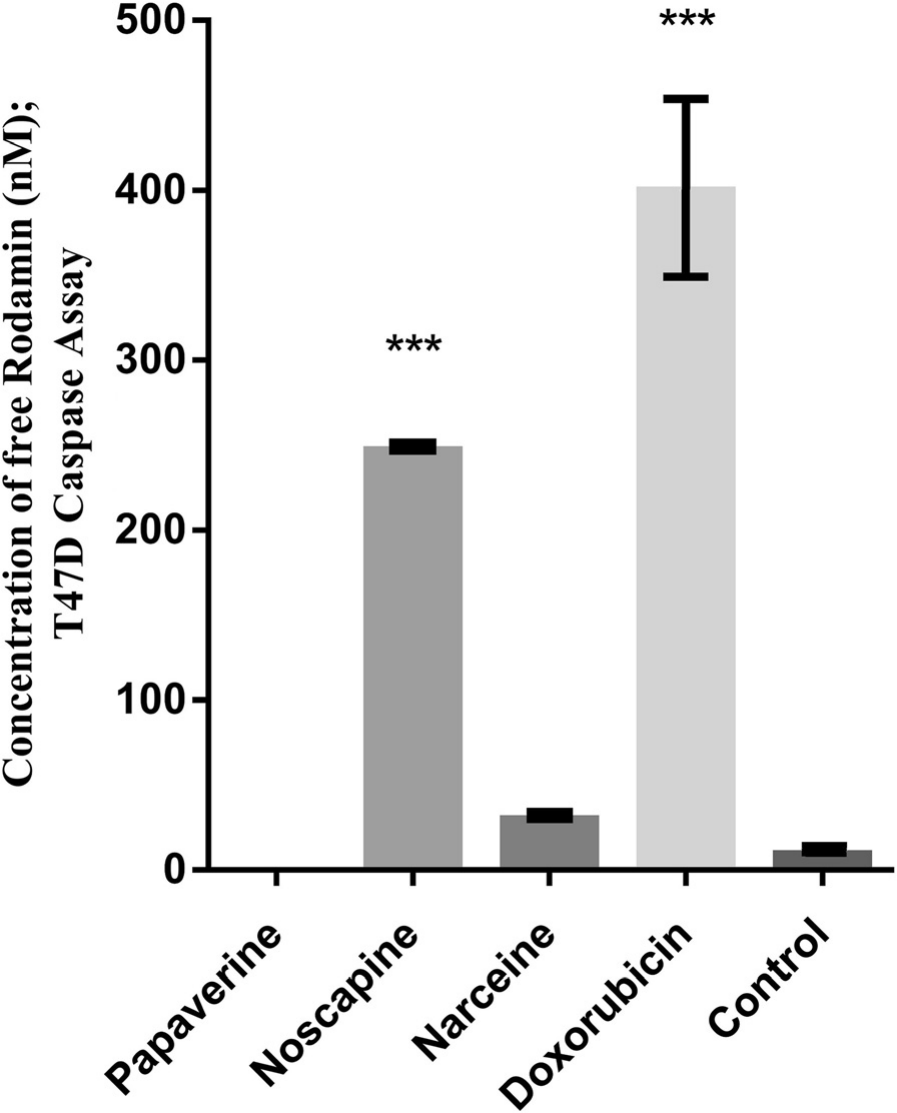
**The activity of caspase-3 after 48 hours incubation of post-treatment T47D cells.** Data are reported as mean ± SD. It is shown significant increase in caspase 3 activities after treatment by doxorubicin and noscapine (p < 0.001).

Caspase-3 activity was measured by the cleavage of the substrate -Rodamin110- fluorimerically and was quantified by calibration curve (Figure 5). According to Figure 5 and in opposite to doxorubicin and noscapine, papaverine did not increase caspase activity.

## Discussion

The present study noted that noscapine and papaverine had dose-dependent cytotoxic effects on cancer cell lines, without any cytotoxic effect on noncancerous NIH-3 T3 cells. Minor, non-significant increases are visible in cytotoxicity curves that maybe related to the nonspecific toxicity caused by high concentrations of all three alkaloids used in this study. Therefore the selective toxicity observed in the present project as well as the unselective doxorubicin-induced toxicity could have affected both cancerous and noncancerous cells. To determine whether or not apoptosis was considered as the major cell death pathway, the cell lines were examined by using annexin -V affinity, Comet assay, DNA laddering and Caspase assay. The annexin V assay has been widely accepted as a marker of apoptosis. Annexin V, a calcium-dependent phospholipid-binding protein, binds to PS residues on the cell membrane that are translocated to the outside of the cell due to apoptosis [28,29]. DNA damage is a hallmark of cell death [30] and alkaline single cell gel electrophoresis (SCGE) or comet assay is a very sensitive assay for detection of some kinds of DNA damage including single strand DNA breakage, alkaline-labile sites and other damage that generates DNA breaks [25,30]. Based on the results of this study, noscapine and papaverine induced DNA damage followed by apoptosis on HT-29 and T47D cell lines without significant effect on NIH-3 T3 as a noncancerous cell line. Apoptotic effect of noscapine was confirmed by the results of DNA fragmentation and caspase assay. It should be mentioned that even though many other studies have previously noted apoptosis induced by noscapine on cancer cells, a limited number of research has focused on noncancerous cell lines [12,31]. Recently, noscapine has been recognized as a kinetic stabilizer compound. Interestingly, noscapine binds to different site of tubulin when compared to other known tubulin-binding agents. Additionally, even though noscapine’s binding to tubulin site is not very strong, it is adequate enough to arrest the cell cycle [31]. Therefore, this feature might explain the selective cytotoxicity of noscapine. Based on the results of the present study and in contrast to noscapine and what was expected, papaverine did not increase caspase activity. Several studies have shown the apoptotic effect of papaverine [17,19,20,32]. However, a study on human promyelocytic leukemia HL60 cells declined cytotoxic and apoptotic effects of this alkaloid [33]. According to the results of DNA damage and apoptotic annexin-V assays, papaverine produced genotoxicity through induction of apoptosis in cancer cells. This lack of caspase activity has been also reported previously by Rubis et al. [20]. The results of the later study as well as our study may indicate that caspase-independent apoptosis pathways are involved in the programmed cell death related to papaverine. Further studies seem to be necessary to better clarify the exact mechanisms of papaverine-dependent apoptosis. Furthermore, in spite of DNA damage in HT-1080 after alkaloids treatment (Figure 2), apoptosis was not confirmed as a cell death pathway in this cell line. This contradiction might be related to intracellular repair system. However, the effects of narceine were different from those seen with the other two alkaloids on the cells in our study. Among the cells tested in this study, only HT-1080 cells were affected by DNA damage induced by narceine. However this damage did not result in apoptosis. It should be mentioned that all the findings about narceine were obtained despite the fact that no cytotoxicity was present when MTT assay was performed. Part of these findings may be related to other pathways of cell toxicity or death. However, more studies are needed to determine anti-tumor activity of narceine.

## Conclusion

In the present study, we demonstrated that noscapine and papaverine inserted selective cytotoxicity effects on cancer cell lines in comparison to doxorubicin which showed unselective cytotoxic effects on both malignant and non-malignant cell lines. This effect may present a new approach for using non-addictive opium alkaloids in the treatment of cancer. Further investigations are needed to clarify and confirm the selective cytotoxic effects of these alkaloids on other noncancerous cell lines.
